# Identification and Characterization of a *Streptomyces* *albus* Strain and Its Secondary Metabolite Organophosphate against Charcoal Rot of Sorghum

**DOI:** 10.3390/plants9121727

**Published:** 2020-12-07

**Authors:** Subramaniam Gopalakrishnan, Rajan Sharma, Vadlamudi Srinivas, Nimmala Naresh, Suraj P. Mishra, Sravani Ankati, Sambangi Pratyusha, Mahalingam Govindaraj, Susana V. Gonzalez, Sondre Nervik, Nebojsa Simic

**Affiliations:** 1 International Crops Research Institute for the Semi-Arid Tropics (ICRISAT), Patancheru 502 324, Telangana, India; s.vadlamudi@cgiar.org (V.S.); n.naresh@cgiar.org (N.N.); S.Suraj@cgiar.org (S.P.M.); sravaniankati@gmail.com (S.A.); s.pratyusha@cgiar.org (S.P.); m.govindaraj@cgiar.org (M.G.); 2 Department of Chemistry, Faculty of Natural Sciences, Norwegian University of Science and Technology (NTNU), 7491 Trondheim, Norway; susana.v.gonzalez@ntnu.no (S.V.G.); sondre.nervik@ntnu.no (S.N.)

**Keywords:** *Macrophomina phaseolina*, charcoal rot, sorghum, biocontrol, organophosphate

## Abstract

*Streptomycesalbus* strain CAI-21 has been previously reported to have plant growth-promotion abilities in chickpea, pigeonpea, rice, and sorghum. The strain CAI-21 and its secondary metabolite were evaluated for their biocontrol potential against charcoal rot disease in sorghum caused by *Macrophomina phaseolina*. Results exhibited that CAI-21 significantly inhibited the growth of the pathogen, *M. phaseolina*, in dual-culture (15 mm; zone of inhibition), metabolite production (74% inhibition), and blotter paper (90% inhibition) assays. When CAI-21 was tested for its biocontrol potential under greenhouse and field conditions following inoculation of *M. phaseolina* by toothpick method, it significantly reduced the number of internodes infected (75% and 45% less, respectively) and length of infection (75% and 51% less, respectively) over the positive control (only *M. phaseolina* inoculated) plants. Under greenhouse conditions, scanning electron microscopic analysis showed that the phloem and xylem tissues of the CAI-21-treated shoot samples were intact compared to those of the diseased stem samples. The culture filtrate of the CAI-21 was purified by various chromatographic techniques, and the active compound was identified as “organophosphate” by NMR and MS. The efficacy of organophosphate was found to inhibit the growth of *M. phaseolina* in the poisoned food technique. This study indicates that *S.* *albus* CAI-21 and its active metabolite organophosphate have the potential to control charcoal rot in sorghum.

## 1. Introduction

Sorghum [*Sorghum bicolor* (L.) Moench] is a nutrient-rich staple food crop for millions of rural communities in the semiarid tropics [1]. It is grown on 42 million hectares of land in the world, with a total production of 59 million tonnes [2]. The productivity of sorghum can be improved if the biotic stresses are well managed. Among the various biotic stresses of sorghum, charcoal rot, caused by *Macrophomina phaseolina* (Tassi) Goid., is an important disease of postrainy sorghum. It affects more than 500 plant species including legumes, vegetables, and cereals and devastates up to 100% of the crop [3].

*M. phaseolina* is a soil-borne root pathogen that naturally forms heat-tolerant sclerotia [4]. The pathogen survives predominantly as small black sclerotia in diseased root and stem debris or soil after the decay of the plant material in which they were formed, which serves as the primary source of charcoal rot infection [5]. Germination of sclerotia from sorghum stalks was found to be only 23% after 16 months in the soil, whereas sclerotia from other host plants are known to survive for up to 15 years [4]. The population of sclerotia in the soil is highly variable, and this variation in the primary inoculum density results in variable charcoal rot incidence in the field [6]. The inoculum density increases in the soil by continuous cultivation of susceptible cultivar in the same field. Under drought and high-temperature conditions, the mycelia of *M. phaseolina* deeply penetrate the host crop in search of nutrients, which leads to structural damage and death of the infected plants [7]. *M. phaseolina* produces a metabolite, phaseolinone, which causes vascular blockage that leads to the death of the whole plant [8,9]. This disease affects large areas of postrainy sorghum production fields where moisture stress is predominant and causes a significant yield loss [10]; therefore, researchers around the world are making various attempts to manage this disease.

The biggest drawback in managing charcoal rot in sorghum is the lack of a high level of host-plant resistance in currently cultivated cultivars. Control of charcoal rot in sorghum is challenging, as no single control measure is fully effective. Advanced sowing date, use of pathogen-free (*M. phaseolina*-free) seed, solarization of soil, and treatment with thiram and carbendazim are some of the control measures usually employed to manage charcoal rot disease but with limited success [11,12]. Biocontrol of this soil and seed-borne pathogen has also been attempted with beneficial microorganisms such as *Bacillus*, *Pseudomonas*, *Enterobacter*, *Trichoderma*, *Acinetobacter*, *Amycolatopsis,* and *Streptomyces* [13,14,15,16,17]. This shows the importance of understanding the sorghum–*M. phaseolina* interactions and identifying more potent biocontrol strategies that could be combined with a moderate level of tolerance in the host to manage this disease.

*Streptomyces albus* strain CAI-21 has been previously reported to have plant growth-promotion (PGP) abilities in chickpea, pigeon pea, rice, and sorghum [18,19,20,21,22,23]. Further, in the preliminary investigation, CAI-21 and its culture filtrates were also found to inhibit *M. phaseolina* in the in vitro studies [18]. Therefore, CAI-21 was well adapted not only in the chickpea, pigeonpea, and rice rhizospheres but also in the sorghum rhizosphere, indicating its ability to survive under various natural conditions and its broad spectrum activities. The main objective of the present study was to screen *S. albus* CAI-21 for its biocontrol potential against charcoal rot disease in sorghum by dual-culture assay, secondary metabolite production assay, blotter paper assay, and greenhouse and field assays and to finally purify and identify the active secondary metabolite of CAI-21 responsible for the inhibition of *M. phaseolina*.

## 2. Results and Discussion

### 2.1. In Vitro Inhibitory Activity of S. albus CAI-21

**Figure 1:**
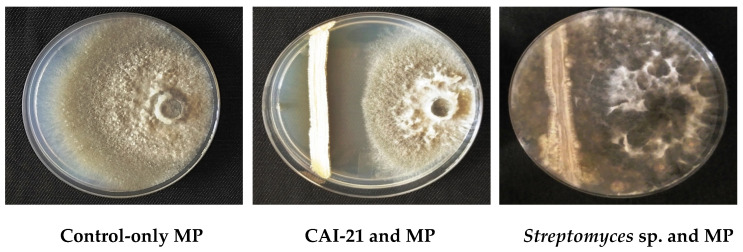
Growth inhibition of *M. phaseolina* (MP) caused by *S. albus* CAI-21 in comparison with non-inhibiting *Streptomyces* strain by dual-culture assay. Both MP and CAI-21 were plated on starch casein agar followed by incubation at 28 ± 2 °C for 120 h in the dark.

**Figure 2:**
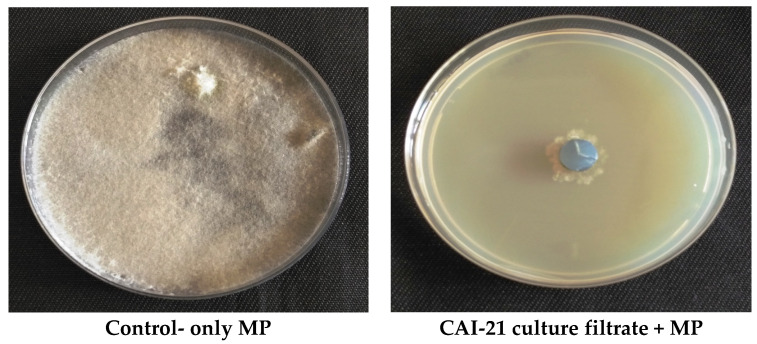
Growth inhibition of *M. phaseolina* (MP) caused by the *S. albus* CAI-21 by secondary metabolite production assay. Left picture: 0.1 mL of methanol was incorporated into the potato dextrose agar (PDA) plate followed by inoculation with 4 mm diameter of MP. Right picture: 0.1 mL of organic fraction in methanol of CAI-21 culture filtrate was incorporated into the PDA followed by inoculation with 4 mm diameter of MP. The plates were incubated at 28 ± 2 °C for 120 h in the dark.

The strain CAI-21 inhibited *M. phaseolina* in both dual-culture and secondary metabolite production assays. In the dual-culture assay, CAI-21 caused a 15 mm inhibition of *M. phaseolina* (Table 1 and Figure 1). This indicated the production of antifungal compounds (such as streptomycin and Actinovate) that may be involved in the inhibition of the hyphal growth of *M. phaseolina*, as there was no direct contact between the *M. phaseolina* and CAI-21. This hypothesis was confirmed in the secondary metabolite production assay as the organic fraction of the culture filtrate of CAI-21 inhibited *M. phaseolina* by 73.8% (Table 1 and Figure 2). Inhibition of *M. phaseolina* growth by PGP microbial strains is mainly attributed to their ability to produce antifungal compounds such as hydrolytic enzymes (cellulase and chitinase) and hydrocyanic acids (HCN) [24,25]. Plant growth-promoting rhizobacteria (PGPR) strains are mainly regarded as biocontrol agents as they potentially produce siderophores, chitinase, and HCN, which induce disease resistance in agriculturally important crops [26,27]. The strain *S. albus* CAI-21 has been previously reported to produce siderophore and HCN [18]. Therefore, it was speculated that *S. albus* CAI-21 may produce one or more secondary metabolites capable of inhibiting *M. phaseolina*.

### 2.2. In Vivo Biocontrol Potential of S. albus CAI-21

**Figure 3:**
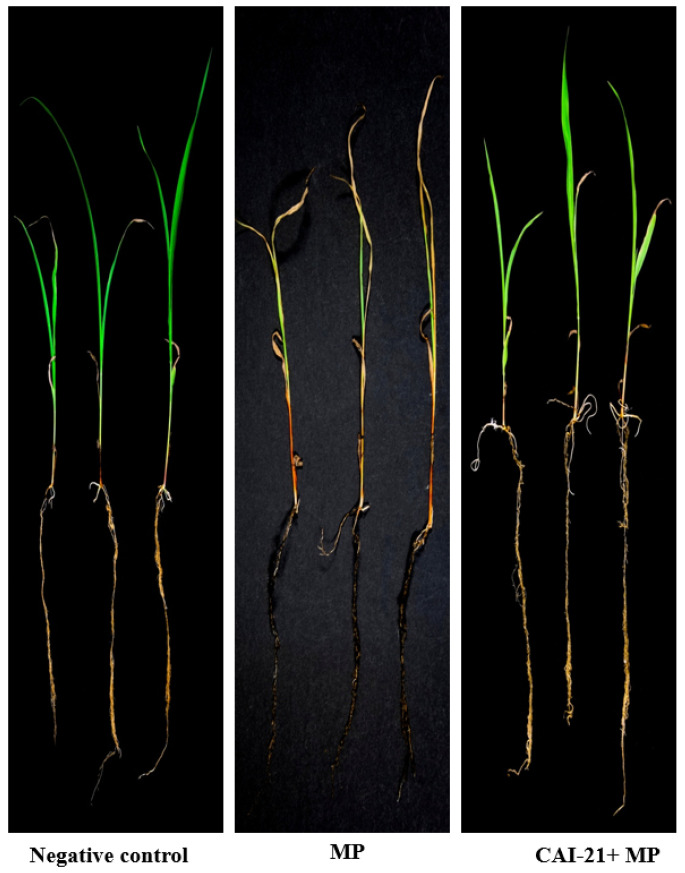
In vivo biocontrol potential of *S. albus* CAI-21 against charcoal rot as determined by blotter paper assay of sorghum roots. MP, *M. phaseolina.*

In the in vivo blotter paper assay, few disease symptoms (score 1 on 0–4 scale rating) and lesser root infections (10–20%) were observed in CAI-21-treated sorghum roots, whereas in the *M. phaseolina*-inoculated control, high disease severity with score 4 and 100% root infection were observed. The negative control plants were found to be healthy as these were without any disease symptoms or root infection (Table 2; Figure 3). Further, the sclerotia of *M. phaseolina* were not found in both in vitro and in vivo studies when treated with CAI-21. Based on both in vitro and in vivo studies, it was concluded that *S. albus* CAI-21 was capable of inhibiting *M. phaseolina*.

### 2.3. Greenhouse and Field Trials

**Figure 4:**
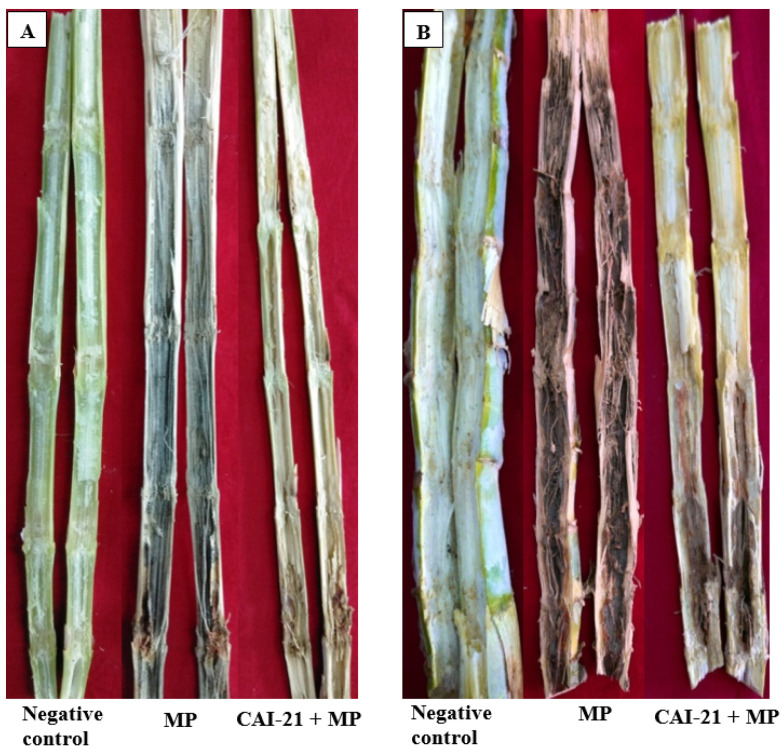
Biocontrol potential of *S. albus* CAI-21 against charcoal rot of sorghum under greenhouse (**A**) and field (**B**) conditions. MP, *M. phaseolina.*

In the greenhouse, when *S. albus* CAI-21 was evaluated for its biocontrol potential against *M. phaseolina* infection, the charcoal rot disease severity was reduced significantly compared to a positive control (only *M. phaseolina* was inoculated). The charcoal rot infection was observed in only 1 internode (75% less) in the CAI-21-treated plants when compared to the positive control, where up to 4 internodes were found to be infected. Further, the length of charcoal rot infection was only 3 cm (75% less) in the CAI-21-treated plants when compared to the positive control, where it was 12 cm (Table 3 and Figure 4A). In the field, the disease severity was significantly reduced in *S. albus* CAI-21-treated plants when compared to the positive control. The charcoal rot infection was observed in only 2.9 internodes in CAI-21-treated plants (48% less), whereas in the positive control, the infection was observed in 5.6 internodes on average. The length of charcoal rot infection was only 10 cm (51% less) in CAI-21-treated plants when compared to the positive control, where it was 19.5 cm (Table 3 and Figure 4B). The reduction of the disease severity could be due to enhanced biotic stress tolerance in the sorghum plants by the CAI-21. PGPR are widely reported for their secondary metabolite production and root-associated hormonal signaling [28]. Charcoal rot of sorghum has been reported to be managed under greenhouse conditions by *Bacillus*, *Brevibacterium*, *Enterobacter*, *Acinetobacter*, *Pseudomonas*, and *Streptomyces* [12,13,18]. However, under field conditions, there is only one report of managing the charcoal rot of sorghum using *Amycolatopsis* [17].

**Figure 5:**
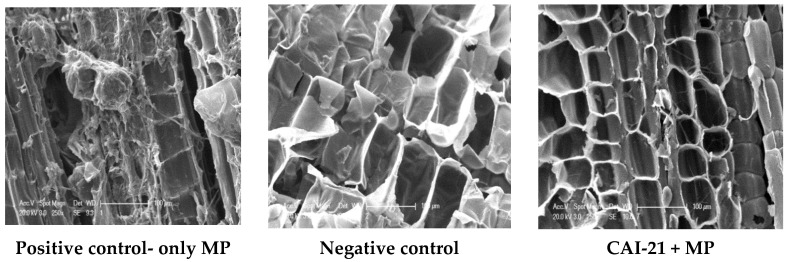
SEM photographs showing morphological changes in the stalks of sorghum of positive control (only MP), negative control, and *S. albus* CAI-21 + MP-treated plants. MP, *M. phaseolina*.

In the present investigation, when the *S. albus* CAI-21- and *M. phaseolina*-treated shoots were observed through the scanning electron microscope, the morphology and size of the phloem and xylem tissues were found to be normal and intact in the *S. albus* CAI-21- and *M. phaseolina*-treated stem samples when compared to positive control, where a major portion of the tissues (>90%) was found to be damaged (Figure 5). The tissues were normal and intact in the shoot samples from the negative control (healthy plants). This implies that *M. phaseolina* was not able to colonize in the CAI-21-treated sorghum plants. Similar observations were noted with *Streptomyces* strains BCA-546 and CAI-8 [13] and *Amycolatopsis* [17]. These results support the hypothesis that the PGPR not only enhance the plant growth through root colonization but also exhibit indirect benefits such as disease suppression as reported in other studies [29,30,31].

### 2.4. Purification of the Active Secondary Metabolite from S. albus CAI-21

**Figure 6:**
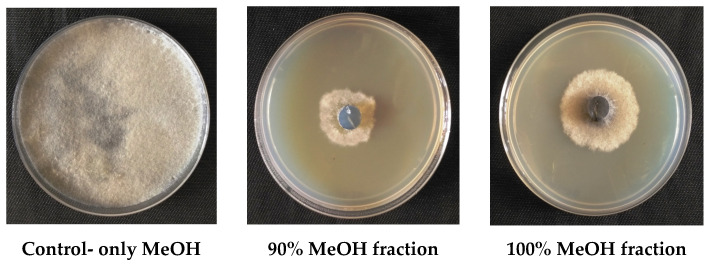
Inhibitory activity of the purified metabolite (90% and 100% MeOH fractions of the open column chromatography) of *S. albus* CAI-21 against *M. phaseolina*.

Rhizospheric microbes often produce a wide variety of secondary metabolites that are known to aid in inhibiting fungal pathogens [32]. In this investigation, *S. albus* CAI-21 consistently inhibited *M. phaseolina* in dual-culture, secondary metabolite production, and blotter paper assays, and its antagonistic potential was further demonstrated under the greenhouse and field conditions. This may be due to the ability of CAI-21 to produce one or more antifungal compounds, and this perhaps prevented *M. phaseolina* colonization and further spread of charcoal rot infection in sorghum plants. To prove this hypothesis, the identification of one or more secondary metabolites responsible for the inhibition of *M. phaseolina* was carried out from the culture filtrates of *S. albus* CAI-21 by various chromatographic techniques. When the culture filtrates of CAI-21 were partitioned three times against ethyl acetate, organic ethyl acetate and aqueous fractions were obtained; however, the antagonistic activity was found only in the organic fraction. The active organic fraction was further purified on open column chromatography packed with C18 and eluted with incremental MeOH. Of all the fractions, only 90% of the MeOH fraction was found to be most active against *M. phaseolina* in growth inhibition followed by 100% of the MeOH fraction (Figure 6). Hence, 90% and 100% MeOH fractions were further purified in HPLC and analyzed through NMR and MS spectroscopy.

### 2.5. Identification of the Active Secondary Metabolite from S. albus CAI-21

Preliminary NMR analyses pointed out that both fractions still contained impurities, which interfered with the sample signals. The impurities were removed on the preparative HPLC, and the purified samples were dissolved in pure methanol. The final purified sample yielded 26 mg of white amorphous powder, which was submitted for further NMR and MS analyses.

**Figure 7:**
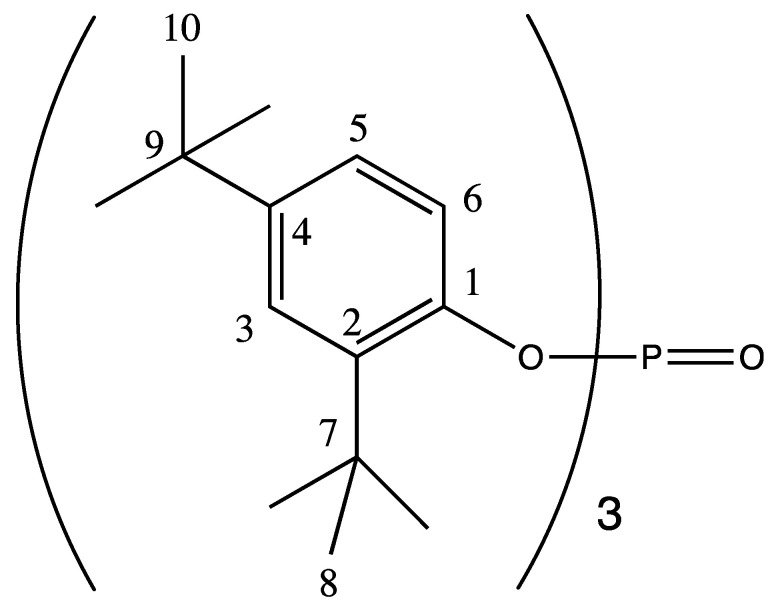
Structure of the identified compound. NMR data: ^1^H NMR (CDCl_3_, 600 MHz, 300 K) δ: 1.28 (bs, 27H, H-10), 1.33 (s, 27H, H-8), 7.12 (dd, 3H, *J* = 2.5/8.6 Hz, H-5), 7.36 (dd, 3H, *J* = 1.9/2.5 Hz H-3), 7.53 (dd, 3H, ^4^*J*_1H,31P_ = 0.8Hz; ^3^*J*_H,H_ = 8.6 Hz, H-6. ^13^C NMR (CDCl_3_, 150 MHz, 300 K) δ: 30.2 (C-8), 31.5 (C-10), 34.5 (C-9), 34.9 (C-7), 119.1 (d, ^3^*J*_13C, 31P_ = 2.0 Hz, C-6), 124.0 (C-5), 124.5 (C-3), 138.5 (d, ^3^*J*_13C, 31P_ = 9.0 Hz, C-2), 147.1 (C-4), 147.7 (d, ^2^*J*_13C, 31P_ = 6.6 Hz, C-1).Organophosphates are well known for their pesticide and insecticide functions in agriculture as they work as a neurotoxin to kill pests [33]. Organophosphates, especially chlorpyrifos, were reported for the control of insect pests of many crops including sorghum such as *Rhyzopertha dominica* (F.), *Tribolium castaneum* (Herbst), *Oryzaephilus surinamensis* (L.), and *Cryptolestes ferrugineus* (Stephens) in combination with bifenthrin and piperonyl butoxide at the concentrations of 300 and 1200 ppm [34,35]. However, their fungicidal role was less explored and not reported in sorghum. The antifungal activity of different organophosphates (1a, 2a–b, 3a–b, and 4a–b) on *Aspergillus niger* and *Fusarium oxysporum* and their antifungal ability that increased with the increasing concentration from 50 to 200 ppm were reported [36]. Furthermore, all the known organophosphates are synthesized chemically and used for pest management. However, their biological synthesis is also reported in cyanobacteria [37] and *Streptomyces*. *Streptomyces* spp. are the major natural microbial source for many bioactive compounds with organophosphorus functional groups [38]. *Streptomyces antibioticus* DSM 1951, *S. lavendulae* NK901093, and *Streptomyces* sp. JP90 were reported to synthesize organophosphates that are potent insecticides [39,40,41]. Apart from organophosphates, which are phosphate esters, their structural analogues phosphonates are also reported from *Streptomyces* origin for insecticidal and herbicidal activities [42]. Similarly, the organophosphate from CAI-21 can be explored for the control of insect pests of sorghum in addition to charcoal rot.

The ^1^H NMR spectrum of the sample showed the presence of a 1,2,4-substituted benzene ring. By combining 1D and 2D NMR data (Figures S1–S4), two substituents were easily identified as *t*-butyl groups, while the third one contained heteroatoms, among which at least one was oxygen. The HR-MS analysis showed a mass of 662.4542 (Figure S5), which corresponded to the molecular formula C_42_H_63_O_4_P. The observed fragmentation pattern suggested a repetition of aromatic unit in the structure. The combined NMR and MS data were consistent with an organophosphate containing three substituted benzene rings (Figure 7).

*Streptomyces* is widely reported to produce secondary metabolites/antibiotics such as streptomycin, Actinovate, blasticidin-S, and validamycin. Among the known antibiotics available on the market, more than 60% of them are produced by the genus *Streptomyces* [43]. Some of the antibiotics produced by the genus *Streptomyces* include streptomycin (by *S. griseus*), Actinovate (by *S. lydicus* WYEC 108), blasticidin-S (by *S. griseochromogenes*), validamycin (by *S. hygroscopicus*), kasugamycin (by *S. kasugaensis*), oxytetracycline (by *S. rimosus*), polyoxins (by *S. cacaoi* var. *asoensis*), Mycostop (by *Streptomyces* sp. K61), azalomycin B (by *Streptomyces* sp. HAAG3-15), abamectin/avermectins (by *S. avermitilis*), emamectin benzoate (by *S. avermitilis*), polynactins (by *S. aureus*), natamycin (by *S. natalensis* and *S. chattanoogensis*), the diketopiperazine cyclo(Tre-Phe) (by *Streptomyces* sp. SAI-25), a novel fatty acid amide derivative (by *Streptomyces* sp. CAI-155), and milbemycin (by *S. hygroscopicus* subsp. *aureolacrimosus*), which are widely reported as crop protection agents [44,45,46,47]. Similarly, the organophosphate secreted by *S. albus* CAI-21 may be used in biological control of *M. phaseolina*.

## 3. Materials and Methods

### 3.1. PGP Microbe

One strain of *Streptomyces albus* CAI-21, previously identified by whole-genome sequencing and reported to have the capacity for PGP traits such as shoot mass, shoot volume, and root mass in sorghum [18,22], was collected from our lab and used in the present study.

### 3.2. In Vitro Dual-Culture and Secondary Metabolite Production Assays

*S. albus* CAI-21 was tested for its inhibitory activity against *M. phaseolina* (isolated from charcoal rot-infected sorghum plants at International Crops Research Institute for the Semi-Arid Tropics (ICRISAT), Patancheru, India) by dual-culture assay as described earlier [18]. In brief, a fungal disk (*M. phaseolina*) of 6 mm diameter was placed on one edge (1 cm from the corner) of the starch casein agar plate, and CAI-21 was streaked on the other edge of the plate (1 cm from the corner), followed by incubation at 28 ± 2 °C for 5 days. Inhibition of fungal mycelium (halo zone) around the CAI-21 was noted as positive, and the inhibition zone (in mm) was measured. Positive control plates contained only *M. phaseolina*, while negative control plates contained only sterile water. For the secondary metabolite production assay, CAI-21 was grown on starch casein broth (SCB; composition—soluble starch 10 g, casein 0.3 g, KNO_3_ 2 g, MgSO_4_.7H_2_O 0.05 g, K_2_HPO_4_ 2 g, NaCl 2 g, CaCO_3_ 0.02 g, FeSO_4_.7H_2_O 0.01 g, and distilled water 1000 mL) at 28 ± 2 °C for seven days. After seven days of incubation, the cell-free supernatant was collected, and its secondary metabolites were extracted using ethyl acetate by the solvent partitioning method [48]. The pellet containing the biomass was discarded. The resultant aqueous and organic fractions were tested against *M. phaseolina* for their growth inhibition ability by the modified poisoned food technique [13]. In brief, potato dextrose agar plates with 0.1 mL of either organic or aqueous fractions were prepared. A fungal disc with 4 mm diameter of *M. phaseolina* was bored and placed at the center of the plate. After five days of incubation at 28 ± 2 °C, the fungal growth was measured and compared with the positive control plates, where only 0.1 mL of methanol was added, and chemical control plates, where only chlorpyriphos (20%) was added. Negative control plates contained only sterile water. Both the dual-culture and the secondary metabolite production assays were repeated thrice to confirm the results.

### 3.3. In Vivo Blotter Paper Assay

CAI-21 was evaluated for its antifungal activity against *M. phaseolina* on two-week-old seedlings of susceptible sorghum line 296B using a blotter paper assay [17]. In brief, a total of 3 treatments (CAI-21 + *M. phaseolina* inoculation; positive control, only *M. phaseolina* inoculation; and negative control, only sterile water) were tested. Fifteen plants were used per replicate, and 3 replications were made for each treatment. The symptoms of charcoal rot (black-colored infection and microsclerotia on the root surface) were recorded on a 0–4 rating scale (0 = no visible disease symptom; 4 = maximum disease symptoms; [49]). The percentage of infected roots on CAI-21-inoculated treatment compared with the positive (only *M. phaseolina* inoculated) control was also calculated. The assay was repeated twice to confirm the results.

### 3.4. Greenhouse Trials

CAI-21 was evaluated for its biocontrol potential against the charcoal rot of sorghum under greenhouse conditions. This was performed with the toothpick inoculation method [17,50]. In brief, a total of 3 treatments (CAI-21 + *M. phaseolina* inoculation; positive control, only *M. phaseolina* inoculation; and negative control, only water) were tested with 10 replications. The trial was conducted in a completely randomized design. A mixture of 2 kg of Vertisols, sand, and farmyard manure (at 3:2:1 ratio) was filled in 8-inch plastic pots. Sorghum seeds (line 296B) were surface-sterilized with 2.5% sodium hypochlorite solution in water for 3 min and rinsed several times with sterilized distilled water. The surface-sterilized seeds were soaked in CAI-21 suspension (at 10^8^ CFU ml^−1^ grown in SCB separately; the CFU was calculated by the plate count method) or in sterilized water (for a negative control) for one hour. The treated seeds (three per pot) were sown immediately at 3 cm depth and thinned to one plant per pot after germination. Booster doses of CAI-21 (5 mL per seedling) were applied by the soil drench method at 15 and 30 days after sowing (DAS). Ten days after flowering, all the plants (except the ones in the negative control) were artificially inoculated by inserting a toothpick infested with the inoculum of *M. phaseolina* into the second internode of the stalk [50]. At harvest, the disease severity was recorded by measuring the number of internodes infected and the length of infection. The greenhouse trial was repeated twice to confirm the results.

At harvest, the stem samples were also examined by scanning electron microscopy (SEM) analysis for colonization of CAI-21 and for any morphological changes that might have occurred [13,51]. In brief, small pieces of the infected portion of the stem were cut, fixed in 2.5% glutaraldehyde in 0.1 M phosphate buffer (pH 7.2) for 24 h at 4 °C, and postfixed in 2% aqueous osmium tetroxide for 4 h. The stem samples were dehydrated with a series of graded alcohols and dried to a critical point with a critical point drying (CPD) unit. The processed samples were mounted over the stubs with double-sided carbon conductivity tape, and the samples were coated with a thin layer of gold using an automated sputter coater (Model—JEOL JFC-1600) for 3 min and scanned using SEM. SEM analysis was performed at RUSKA Lab, College of Veterinary Science, Rajendranagar, Hyderabad, India. The morphological changes of the cells were measured using SEM at the required magnifications.

### 3.5. Field Trials

CAI-21 was also evaluated for its biocontrol potential against charcoal rot of sorghum under field conditions at International Crops Research Institute for the Semi-Arid Tropics (ICRISAT), Patancheru, India, during the postrainy 2017−2018 cropping season by toothpick inoculation method, as described in greenhouse trials. During the cropping season, a minimum nighttime temperature range of 11–17 °C and a maximum daytime temperature range of 28–38 °C were recorded. The trial was conducted in a randomized complete block design (RCBD) with three replications. The plot size was 3 rows of 2 m long with a row-to-row spacing of 75 cm and a plant-to-plant spacing of 10 cm. Sorghum seeds (line 296B) were sown in the field at a depth of 5 cm to achieve an estimated plant stand density of 60 plants per replication. A total of three treatments (CAI-21 + *M. phaseolina* inoculation; positive control, only *M. phaseolina* inoculation; and negative control, only water) were evaluated. Just before sowing, the seeds of 296B were treated with CAI-21 (containing 10^8^ CFU mL^−1^) for 50 min and sown by hand. CAI-21 (1000 mL; 10^8^ CFU mL^−1^) was also applied every 15 days in the soil close to the plant until the flowering stage. Plants were artificially inoculated with the *M. phaseolina*, by inserting a toothpick infested with *M. phaseolina* into the second internode of the sorghum stalk 10 days after 50% flowering. The growth of the *M. phaseolina* on toothpicks was achieved as described earlier [50]. Irrigation was withheld in the experimental plots at 50% flowering to ensure adequate soil moisture stress to facilitate disease development. At the physiological maturity stage (~35 days after inoculation of *M. phaseolina*), the disease severity was recorded by measuring the length of infection (in cm) and the number of internodes infected.

### 3.6. Purification and Identification of Active Metabolite from S. albus CAI-21

CAI-21 was grown on SCB at 28 °C for 7 days in an orbital shaker at 120 rpm. At the end of the 7-day incubation, the culture was centrifuged at 12,000× *g* for 15 min at 4 °C, and the supernatant (cell-free culture filtrates) was collected. The culture filtrates (10 L) were partitioned three times against ethyl acetate (EtOAc; one-third of the volume each time), and the resultant organic (EtOAc) and aqueous fractions were evaporated and collected in methanol (10 mL). Both organic and aqueous fractions were tested against *M. phaseolina* by a modified poisoned food technique [13].

The organic fraction, which showed antagonistic potential, was subjected to C18 open column chromatography. The column (23 × 3.6 cm) was packed with C18 (Wakosil 40C18, Wako, Osaka, Japan) one day prior to allow the C18 to settle in the column. After conditioning the C18 column, the bioactive fraction was loaded and eluted with a gradient of methanol in Milli-Q water (5%, 10%, 20%, 40%, 60%, 80%, and 100% MeOH). All the collected fractions were evaluated for their antagonistic activity against *M. phaseolina* by the poisoned food technique [13].

The most bioactive open-column chromatography fraction was further purified on HPLC (Water alliance 2695 separation module, Waters, Milford, Massachusetts, USA; UV detector/254 nm; Atlantis C_18_ analytical column, 5 µm, 250 × 4.6 mm, flow rate 0.5 mL min^−1^; gradient method with acetonitrile/water) and subjected to structural identification studies. Removal of impurities in the purified sample was performed with a preparative HPLC Agilent 1260 Infinity instrument, Santa Clara, USA equipped with two G1361A 1260 Prep Pumps, a G2260A 1260 Prep ALS, G1316A 1260 TCC Switching Valve, a G7114A 1260 Variable Wavelength Detector, and a G7102 1290 Evaporative Light Scattering Detector. An Agilent 5 Prep-C18 (21.2 × 150 mm, 5 µm) column was used with a flow of 20 mL/min. Samples were dissolved in pure methanol and eluted isocratically in methanol/water 80:20 for 4.5 min and then isocratically in pure methanol for 8.5 min. The fraction at t_R_ = 11.64 min was collected in a G1364B 1260 FC-PS fraction collector and evaporated in a smart evaporator under a stream of nitrogen.

### 3.7. Characterization of the Purified Compound

#### 3.7.1. Mass Spectrometry (MS)

An accurate mass determination was carried out on a “Synapt G2-S” Q-TOF instrument from Waters™ (Milford, Massachusetts, USA). Samples were ionized using an ESI (Electrospray ionization) probe in positive mode. No chromatographic separation was used previously for the mass analysis. The calculation of the exact mass and spectral processing was performed using Waters™ MassLynx V4.1 SCN871 software.

#### 3.7.2. Nuclear Magnetic Resonance (NMR)

NMR data were acquired using a Bruker Avance III HD spectrometer, operating at a proton frequency of 600.18 MHz, with a 5 mm triple-resonance cryogenic CP-TCI probe, and equipped with a z-gradient. The samples containing a solution of 15 mg of the substance in chloroform-d (CDCl_3_) were measured at 300 K, using TMS signal as a reference. The following 1D and 2D pulse sequences from the Bruker User Library were used for the NMR experiments: ^1^H 1D (600 MHz), ^13^C 1D (150 MHz), 2D heteronuclear single quantum coherence (HSQC) with multiplicity editing, 2D heteronuclear multiple bond correlation (HMBC) with threefold low-pass *J*-filter to suppress one-bond correlations, and 2D H–H-correlation spectroscopy (COSY) with gradient pulses for selection.

### 3.8. Statistical Analysis

The data were analyzed statistically by analysis of variance (ANOVA; Genstat 20. version) in a completely randomized design (CRD) for greenhouse and randomized complete block design (RCBD) for field studies to evaluate the efficiency of *S. albus* CAI-21. The mean values for both greenhouse and field studies were compared at 5% level of significance.

## 4. Conclusions

It is concluded from the results of this study that CAI-21 may be the first *Streptomyces* strain to be reported to manage the charcoal rot of sorghum in both greenhouse and field experiments. Furthermore, the active metabolite responsible for the inhibition of *M. phaseolina* was identified as an organophosphate. In the absence of a high level of genetic resistance in high-yielding varieties, CAI-21 and its metabolite could be effective in managing charcoal rot disease in sorghum. Thus, this strain may be used as an important tool in managing charcoal rot in sorghum. Further experiments are needed to determine the effectiveness of the CAI-21 and the organophosphate, as an effective antifungal compound, under different field conditions and using diverse sorghum cultivars. The biosafety issues related to the organophosphate secreted by the CAI-21, such as risk assessment for human and domestic pet health and environmental contamination, also need to be assessed before it is released in the environment.

## Figures and Tables

**Table 1:** In vitro inhibitory activity of *S. albus* CAI-21 against *M. phaseolina*—dual-culture assay and secondary metabolite production assay.

Treatments	Dual-Culture Assay ^@^	Secondary Metabolite Production Assay ^#^
CAI-21 + MP	15	73.8
Positive control (only MP)	0	0.0
Negative control (only sterile water)	0	0.0
Chlorpyrifos 20%	-	68.8
SD	6.8	37.2
LSD (5%)	0.99	2.16

^@^, zone of MP growth inhibition in mm; ^#^, percentage of MP growth inhibition by 10% of the organic fraction of the CAI-21 cell-free extract; -, not done; MP *M. phaseolina*; SE, standard error; LSD, least significant difference; SD, standard deviation.

**Table 2:** Evaluation of *S. albus* CAI-21 for its biocontrol potential against charcoal rot of sorghum—blotter paper assay.

Treatments	Blotter Paper Assay ^	
	Visual Rating	Root Infection (%)
CAI-21 + MP	1	10
Positive control (only MP)	4	100
Negative control (only sterile water)	0	0
SD	1.8	47.7
LSD (5%)	0	0

MP, *M. phaseolina*; ^, the symptoms of charcoal rot were noted in the 0–4 rating scale (0 = no visible symptom; 4 = maximum symptoms). The percentage of infected roots on CAI-21-inoculated treatment compared with positive control was also calculated; SE, standard error; LSD, least significant difference; SD, standard deviation.

**Table 3:** Evaluation of *S. albus* CAI-21 for its biocontrol potential against charcoal rot of sorghum under greenhouse and field conditions.

	Greenhouse		Field	
Treatments	Number of Internodes Infected	Length of Infection (cm)	Number of Internodes Infected	Length of Infection (cm)
CAI-21 + MP	1.0 **	3.0 **	2.9 ***	10.0 ***
Positive control (only MP)	4.0	12.0	5.6	19.5
Negative control	0.0	0.0	0.0	0.0
SD	1.8	5.5	2.4	8.4
LSD (5%)	1.24	3.29	1.36	5.04

MP, *M. phaseolina*; **, statistically significant at 0.01; ***, statistically significant at 0.001; LSD, least significant difference; SD, standard deviation.

## Supplementary Materials

The following are available online at https://www.mdpi.com/2223-7747/9/12/1727/s1, Figure S1: Proton NMR spectrum; Figure S2: 13C NMR spectrum, Figure S3: 2D HSQC spectrum, Figure S4: 2D HMBC spectrum, Figure S5: MS TOF spectrum with identified M+H ion at m/z 663.4540.
