# Supplementary material for: Identification and Characterization of a Streptomyces albus Strain and Its Secondary Metabolite Organophosphate against Charcoal Rot of Sorghum
Source: Plants (Basel). 2020 Dec 7;9(12):1727. doi: 10.3390/plants9121727 (PMC7762395; doi:10.3390/plants9121727)
Supplement: Supplementary file 1 [file plants-09-01727-s001.pdf]

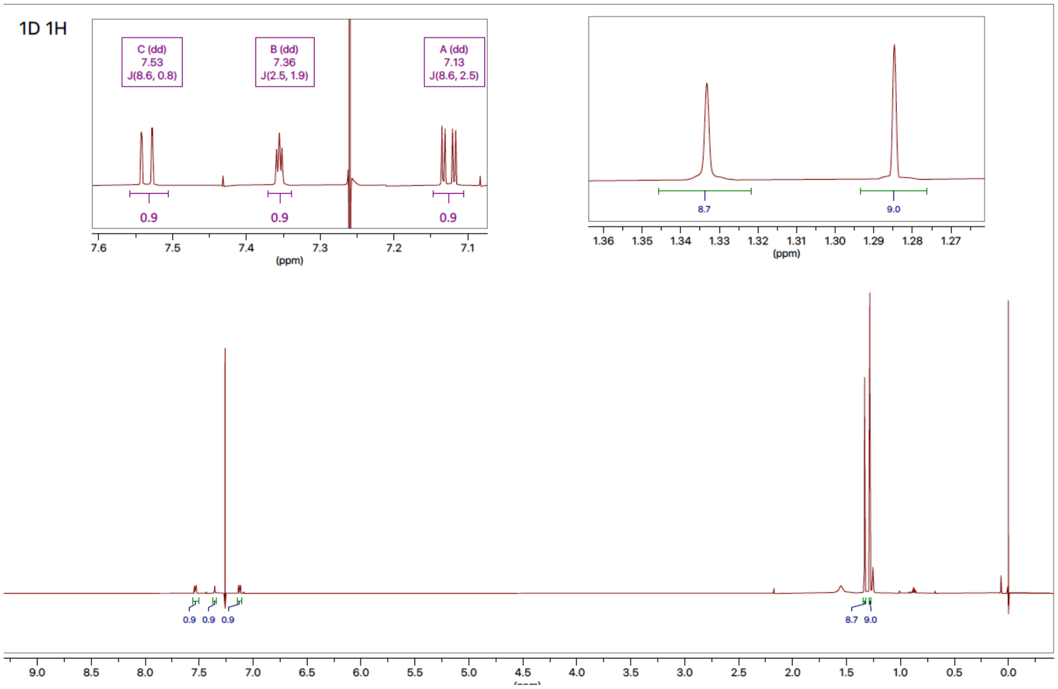

Supplementary Fig. 1. Proton NMR spectrum

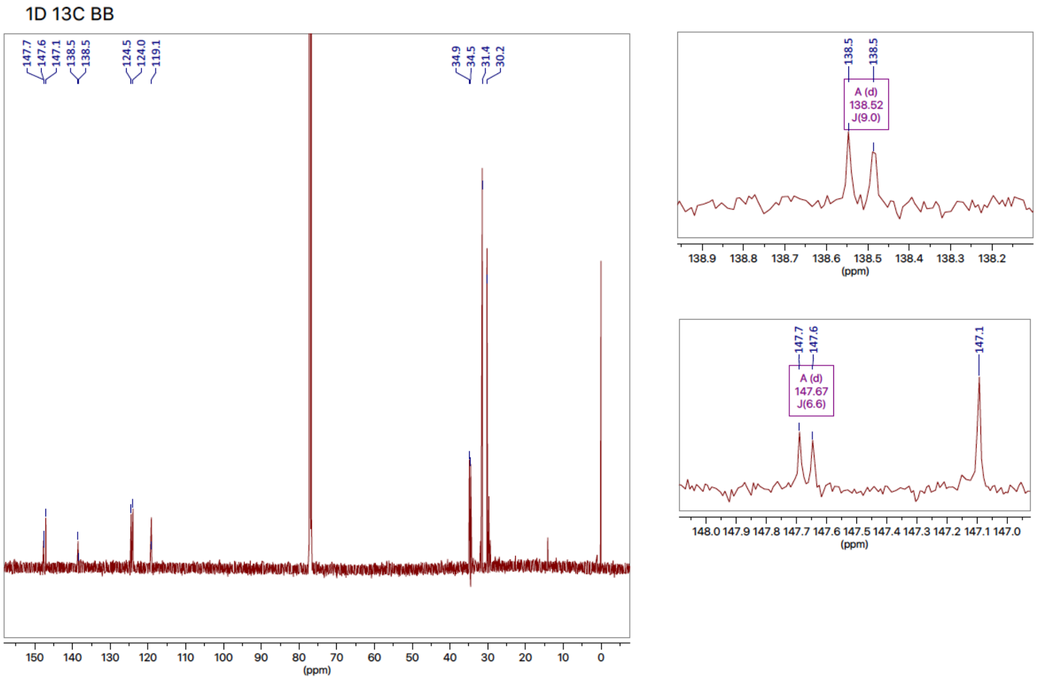

Supplementary Fig. 2. ^{13}C NMR spectrum

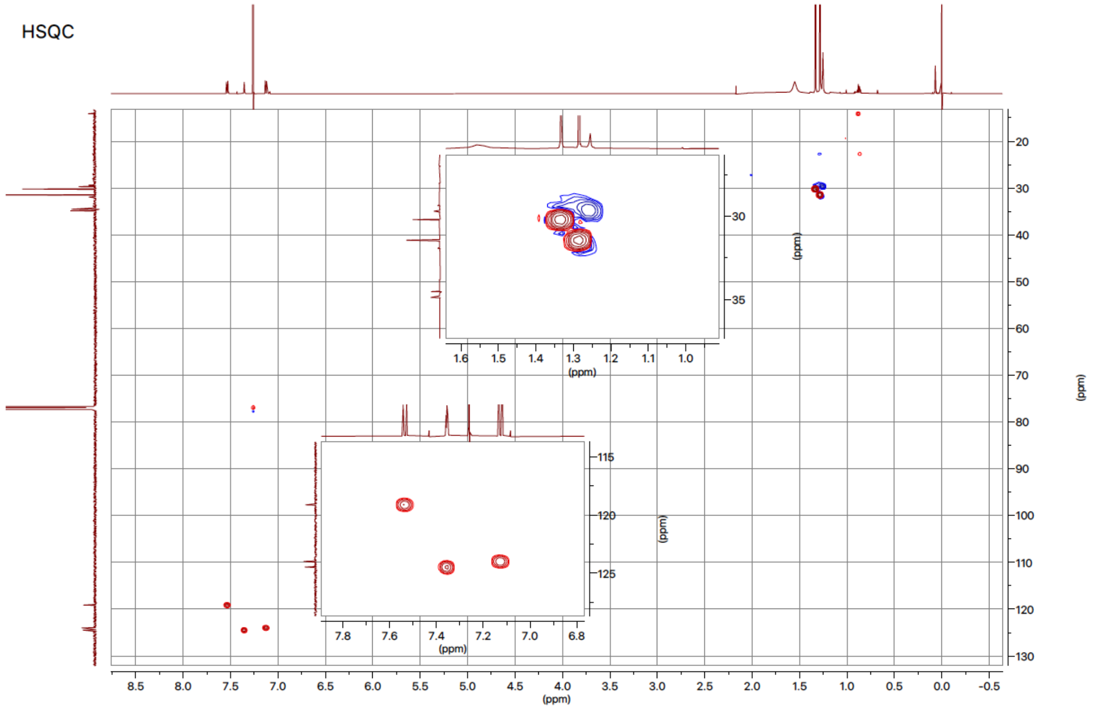

Supplementary Fig. 3. 2D HSQC spectrum

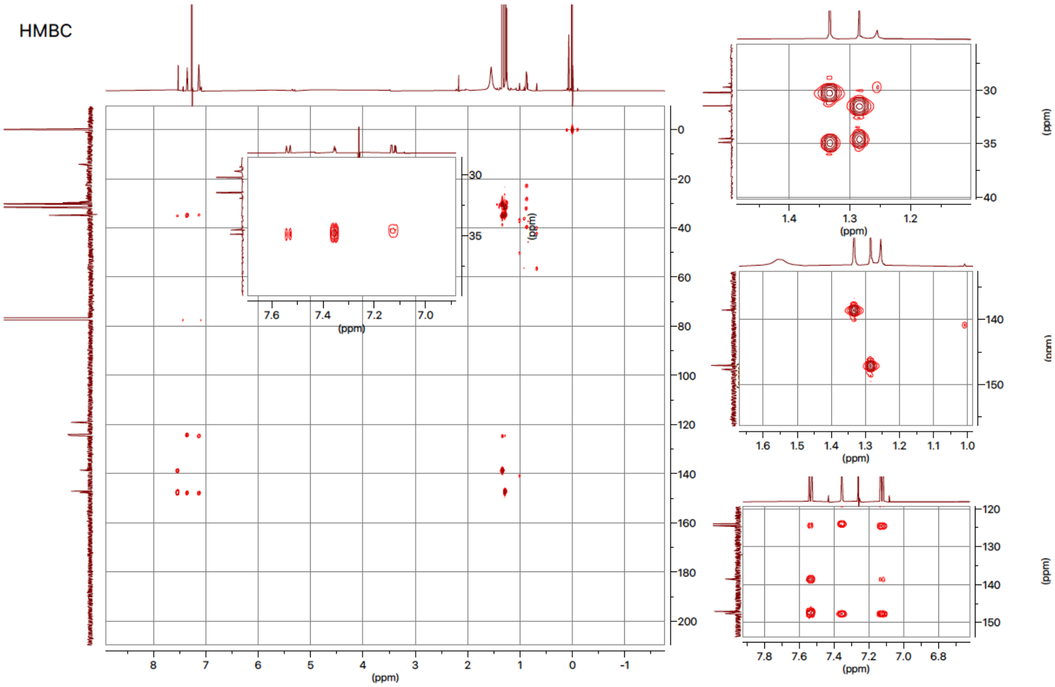

Supplementary Fig. 4. 2D HMBC spectrum

Elemental Composition Report

Single Mass Analysis

Tolerance = 2.0 PPM / DBE: min = -2.0, max = 50.0
Element prediction: Off
Number of isotope peaks used for i-FIT = 4

Monoisotopic Mass, Even Electron Ions
5383 formula(e) evaluated with 5 results within limits (up to 50 closest results for each mass)
Elements Used:
C: 1-500 H: 0-1000 N: 0-10 O: 0-10 P: 0-2 Au: 0-3

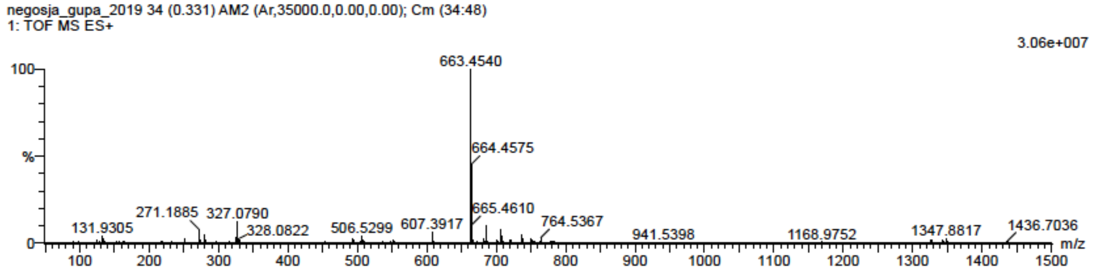

Minimum:				-2.0				
Maximum:		5.0	2.0	50.0				

Mass	Calc. Mass	mDa	PPM	DBE	i-FIT	Norm	Conf(%)	Formula
663.4540	663.4542	-0.2	-0.3	11.5	1361.5	0.001	99.91	C42 H64 O4 P
	663.4544	-0.4	-0.6	3.5	1370.4	8.916	0.01	C32 H63 N4 O10
	663.4534	0.6	0.9	-0.5	1376.6	15.148	0.00	C26 H64 N8 O9 P
	663.4532	0.8	1.2	7.5	1368.6	7.146	0.08	C36 H65 N4 O3 P2
	663.4528	1.2	1.8	1.5	1374.6	13.108	0.00	C30 H62 N2 O Au

Supplementary Fig. 5. MS TOF spectrum with identified M+H ion at m/z 663.4540
